# Archaea on Human Skin

**DOI:** 10.1371/journal.pone.0065388

**Published:** 2013-06-12

**Authors:** Alexander J. Probst, Anna K. Auerbach, Christine Moissl-Eichinger

**Affiliations:** Institute for Microbiology and Archaea Center, University of Regensburg, Regensburg, Germany; University of Waterloo, Canada

## Abstract

The recent era of exploring the human microbiome has provided valuable information on microbial inhabitants, beneficials and pathogens. Screening efforts based on DNA sequencing identified thousands of bacterial lineages associated with human skin but provided only incomplete and crude information on Archaea. Here, we report for the first time the quantification and visualization of Archaea from human skin. Based on 16 S rRNA gene copies Archaea comprised up to 4.2% of the prokaryotic skin microbiome. Most of the gene signatures analyzed belonged to the Thaumarchaeota, a group of Archaea we also found in hospitals and clean room facilities. The metabolic potential for ammonia oxidation of the skin-associated Archaea was supported by the successful detection of thaumarchaeal *amoA* genes in human skin samples. However, the activity and possible interaction with human epithelial cells of these associated Archaea remains an open question. Nevertheless, in this study we provide evidence that Archaea are part of the human skin microbiome and discuss their potential for ammonia turnover on human skin.

## Introduction

Archaea have long been thought of as an ancient form of microorganisms, restricted to extreme environments. However, the picture of Archaea changed within the last decade, when these organisms were found in high abundance in cold and moderate environments all around the world [1].

Archaea might also play an important role in the human body, as methanogenic archaea can contribute up to 12% of total anaerobes in the human gut [2]. In the oral cavity, methanogens have been associated with some periodontal diseases [2], although pathogenesis of an archaeon is yet to be confirmed.

The Human Microbiome Project, founded to decipher the entire set of microorganisms associated with the human body, continues to provide valuable information on how microbial diversity correlates with the health status of humans [3]. So far, the bacterial dynamics of the largest human organ, the skin, have been studied in detail [4], while only two studies report the detection of Archaea on human skin [5], [6]. Hulcr and co-workers studied 60 navels and found three different phylotypes of Archaea appearing marginally in a large subset of bacterial sequences. The archaeal phylotypes identified belonged to the Euryarchaeota and were retrieved from 6 samples only. The authors detected halophiles (*Halobacteriaceae*) in two samples and methanogens (*Methanobrevibacter*) in five different samples. Two of the three archaeal phylotypes were retrieved from a human subject that had not showered for years implying that Archaea are only a minor fraction of the navel and skin microbiome. Moreover, the detected archaeal taxa have previously been found associated with human oral cavity and the human gastrointestinal tract and are thus likely to be oral or fecal contaminants [7], [8]. Although Hulcr and co-workers claimed to be the first to report on Archaea in the human skin microbiome, Caporaso *et al.* [6] had already reported signatures of Archaea, in particular Thaumarchaeota, in samples taken from palms of two individuals. However, they identified these microorganisms as a minor, transient part of the human-associated microbiome and assumed an insignificant role.

Nevertheless, both studies detected Archaea via a co-amplification of their 16 S rRNA genes along with Bacteria. This, and the fact that the primer pairs used did not perfectly match (thaum-)archaeal 16 S rRNA genes, does not allow a conclusion about the role, abundance or diversity of Archaea on human skin. For instance, primer pair F515/R806 (used in [6]) hits only 50% of all Archaea and in particular, 8% of all Thaumarchaeota without mismatch. No perfect match was revealed within the soil crenarchaeotic group (I.1b), which includes *Candidatus* Nitrososphaera (SILVA TestPrime [9]).

The explorations of microbiomes in man-made environments such as clean room facilities, which are strongly influenced by the human (skin) microbiome, have revealed Archaea to be continuously present [10], [11]. Most of these Archaea were Thaumarchaeota, a recently proposed phylum including designated ammonia oxidizers [12]. Due to the recent re-classification of the thaumarchaeal phylum and therefore an assignment of certain crenarchaeal groups to the thaumarchaeal clade, a recent study by LaDuc and co-workers [13] wrongly claimed first evidence of Thaumarchaota in clean room environments, although this group had been identified earlier [10], [11]. So far, these clean room Archaea belonged mainly to the I.1b thaumarchaeal clade, whose representatives are commonly found in the soil microbiome, where they likely contribute to the global nitrogen cycle [14]. A probable association with humans was discussed by Moissl-Eichinger [11] but the question if these Archaea originate from human skin remained unanswered.

In the present study we tackle the question, whether the human skin can be carrier or even habitat for Archaea. We show, that Archaea, and in particular Thaumarchaeota represent a detectable part of the human skin microbiome and their signatures are closely related to those found in hospitals and clean room facilities. Moreover, we provide insight into cell morphology and functional genes for Archaea on human skin.

## Materials and Methods

***Human skin samples*** were taken and handled with approval by and in accordance with the Ethic Commission at the University of Regensburg. The Ethics Commission stated that no ethical concerns are raised by the methods applied and approved the following procedures. Verbal informed consent was obtained from all study participants, which was in agreement with the Ethic Commission’s statement. Each participant handed over the sample right after self-sampling and verbal consent was documented manually along with receiving the samples. Samples were treated anonymously. Human material was not subject of this study. Microbial samples or data derived cannot be attributed to a certain person.

Samples from the entire front torso were taken using DNA-free wipes by the volunteers themselves. The human subjects were instructed to thoroughly wipe their torso (holding the DNA-free wipe with a sterile glove) before taking a regular shower. The volunteers did not apply cosmetics before sampling. The wipes were immediately stored on ice or frozen before processing. An overview of all human skin wipe-samples is given in Table S1.

***Sampling of indoor environments*** was performed with either Biological Sampling Kits (BiSKit, QuickSilver Analytics, Abingdon, MD, USA, according to manufacturer’s instructions) or with a pre-moistened, DNA-free wipe attached to a DNA-free sampling tool made of steel (Table S1). One clean room complex (EADS, Friedrichshafen, Germany) with an ISO 5 and an ISO 8 clean room was sampled. The clean rooms were under certified, fully operating conditions. Additional sampling locations were two intensive care units, one in Regensburg (Germany) and one in Graz (Austria), both maintained fully operating (Table S1).

***Sample extraction from wipes*** (including vortexing and sonication) was performed in 40 ml of PCR grade water (for molecular analyses) or phosphate buffered saline (PBS-buffer, for fluorescence *in situ* hybridization) as described elsewhere [11]. Liquids were concentrated to 200–500 µl using Amicon 50 filter tubes (Millipore, Billerica, MA, USA) before processing.

***The recovery efficiency*** of sampling devices is strongly dependent on the sampling tool and the porosity of the surface [15], [16]. Wipe sampling of non-porous surfaces has been proven to have generally low recovery efficiencies of 8–20% [16], [17]. The fact that human skin is a very porous surface and the sampling of skin was performed by non-specialists (self-sampling) combined with the low recovery efficiency of sampling in general, allows the conclusion that only a small part of the skin microbiome was recovered. However, the ratio of Bacteria and Archaea retrieved is expected to be independent from sampling efficiency.

***Propidium monoazide treatment*** on selected samples from the intensive care unit in Regensburg was performed as described elsewhere [18] prior to DNA extraction. Propidium monoazide (PMA) is a chemical that intercalates to accessible DNA molecules in a given solution and forms a covalent bond after photoactivation of the azide. After PMA-binding the DNA is masked and no longer available for PCR amplification. Cells with intact cell membranes are not penetrated by PMA, their DNA remains unlabeled for PCR amplification and can therefore be detected. This assay allows distinction between the membrane-compromised and the viable microbial community.

***DNA extraction*** was performed by a combination of bead-beating and the XS-buffer method described in Moissl-Eichinger [11], a protocol adapted for low-biomass environments. Bead-beating was included before the application of the XS-buffer to ensure that also hardy microbes were lysed (bead-beating tubes were taken from the MO BIO Power Biofilm™ DNA Isolation Kit, MO BIO, Carlsbad, CA, USA). After bead-beating, beads were washed with 400 µl pure PCR-grade water to decrease sample loss.

***Quantitative PCR of bacterial and archaeal 16 S rRNA gene sequences*** was performed in triplicate as described elsewhere with primer pairs 338 bf/517 ur and 344 af/517 ur, respectively [11], [19] (final primer concentration: 300 nM). 16 S rRNA genes from genomic DNA of the archaeal and bacterial reference strains *Methanosarcina barkeri* and *Bacillus safensis* were amplified with the primer sets 8 af/1406 ur and 9 bf/1406 ur [20], [21]. Quantification of standards was performed with the Qubit Quantitation Platform 2.0 (High Sensitivity Kit, Invitrogen, Carlsbad, CA, USA). Forty cycles of qPCR (Quantitect SYBR Green PCR Mix, Qiagen, Hilden, Germany) were run (RotorGene 6000 Real-Time PCR system, Corbett Life Science, Concorde, NWS, Australia) with an initial denaturation at 95°C for 15 min and a cycling protocol as follows: denaturation at 94°C for 15 sec, annealing at 60°C for 30 sec and elongation at 72°C for 30 sec. Melting curve was performed at 72–95°C. The qPCR efficiencies ranged from 0.87 to 0.92 and R^2^ values of standard curves were in the range of 0.98 to 1.00. Detection limits were defined as 105 copies/µl for archaeal qPCRs (threshold for non-template controls) and 334 copies/µl for bacterial qPCRs. These thresholds were generated as follows: archaeal negative control qPCR revealed minor signals from primer-dimerization, which was confirmed via gel electrophoresis. To exclude such background, qPCR negative controls were averaged and used as a threshold. No archaeal signal was obtained from non-template and extraction controls. Since the qPCR reagents were not free of bacterial DNA as reported elsewhere [22], the detection limit for bacterial qPCR was increased accordingly (334 copies/µl, averaged bacterial qPCR no template controls).

***Amplification of archaeal 16 S rRNA genes*** from samples was performed either directly (primer pair 344 af/915 ar; [23], [24]) or using nested PCR (primer pairs 8 af/UA1406 R [25], [26] and 340 af/915 ar [27], [23]; 2×35 cycles). Primer coverages were evaluated using TestProbe, the SILVA probe match and evaluation tool against the entire SILVA SSU Ref database 108 [28], which revealed those primer combinations with the highest coverage over other combinations studied. The tested combinations included the following primers. Forward: 8af [20], 27FLP [29], 21af [30], 340af [27], 344af [31], A751f [26]. Reverse: 909r [32], 915r [24], 1000R [27], 1100R [33], 1119ar [34], UA1406r [26], 1391r [32], 1406ur [21], 1492ur [21].

***Cloning of 16 S rRNA gene PCR products, sequencing and analysis*** was performed as follows: PCR products were cloned in TOPO10 competent cells (Invitrogen, TOPO® TA Cloning® Kit, pCR®2.1 vector), inserts of positive clones were screened using two (*Hinf*I; *Bsu*RI) and four (*Alu*I; *Hha*I; *Hinf*I; *Rsa*) restriction enzymes after PCR amplification with the above mentioned primer pairs. Clones carrying unique inserts (52 unique patterns) were Sanger-sequenced (100 inserts sequenced), trimmed, quality checked (chimera slayer), aligned [28] and grouped at 1% difference level with the mothur software package [35] and are referred to as operational taxonomic units (OTU) in the manuscript. Representative sequences from each OTU were then classified using the Bayesian classifier [35] against a manually curated GreenGenes database that contains representatives of 98% identical clusters and an updated taxonomy [36] (available at http://www.secondgenome.com/go/2011-greengenes-taxonomy/). The derived archaeal 16 S rRNA gene sequences have been submitted to GenBank (Acc. No. JX865653-JX865767).

***Phylogenetic tree*** of representative OTU sequences (SINA aligned; 28) was computed using ARB [37] and the SILVA database release SSU111 [38] by applying a maximum-likelihood algorithm. Sequences were trimmed to the same alignment length before tree calculation (*E. coli* position 346–943). The tree topology confirmed the classification gained from the Bayesian method (see above).

***Detection of amoA genes*** was performed as described in Tourna *et al.*, 2011 [39] using the primers amoA104F-1d (GCA GGA GAC TAC ATM TTC TA) and amoA616R (GCC ATC CAT CTG TAT GTC CA). Amplicons were cloned and Sanger sequenced using M13 primers. A combined sample of all human subjects investigated in this study was used as a template for PCR (5 µl per 20 µl PCR reaction). Thirty-two sequences were obtained, 28 were of high quality (good chromatogram quality, >800 bps) and 21 of them were identified to be *amoA* genes via BLAST [40] against the NCBI database (deposited under accession numbers KC582378-KC582398). Seven sequences (of approximately 510 bps) showed high similarity to sequences from *Staphylococcus epidermidis* (lipase precursor, *gehD*) and were not included in the analysis. *AmoA* genes were clustered at 97% similarity using mothur [35] and a maximum likelihood tree was computed in ARB with the aid of the *amoA* database from Pester *et al.*, 2012 [37].

***Molecular analysis controls*** for each single step (sampling, DNA extraction, PCR setup) and at least one blank control was carried along, which underwent all detection procedures including regular archaeal PCR, nested-PCR, qPCR, and bacterial qPCR. All Archaea-directed controls were negative, no visible band occurred in any PCR amplification applied. BiSKit blanks exhibited a certain amount of detectable bacterial 16 S rRNA genes (samples from intensive care unit Graz and clean room Friedrichshafen; archaeal control was negative). Therefore all human samples and those from the intensive care unit in Regensburg were taken with DNA-free wipes (dry-heat treated for 24 hrs, 170°C). All wipe extractions blanks were negative in every archaeal and bacterial (q)PCR.

***Fluorescence in situ hybridization (FISH)*** was performed on one human skin sample. The sample was fixed in paraformaldehyde (3% (w/v), final concentration). FISH was conducted as described earlier [41], using probes ARC915 (directed against Archaea, rhodamine green labeled) and EUB338/I (directed against Bacteria, CY3 labeled), 20% (v/v) formamide and 0.01% (w/v) SDS. DAPI was used as counterstain. A NONEUB-Probe was used as a nonsense negative control. Microscopy was performed using an Olympus BX53 microscope (Olympus, Hamburg, Germany; camera: Olympus XM10, software: CelSens Standard 1.5). Fluorescence microscopy was performed using the following filters. U-FUN (excitation 360–370 nm, emission 420 nm IF) for DAPI, U-MINB3 (excitation 470–495 nm, 510 nm IF) for rhodamine green, and U-FRFP (excitation 535–555 nm HQ, emission 570–625 HQ) for CY3. FISH samples in general exhibited a high amount of particles (also from the wipe, see extraction procedure). These particles were either fluorescence active with CY3 filter or with all three filters, clearly distinguishable from microorganisms and not included in the analysis. These fibers made quantitative FISH not feasible.

## Results and Discussion

**Figure 1:**
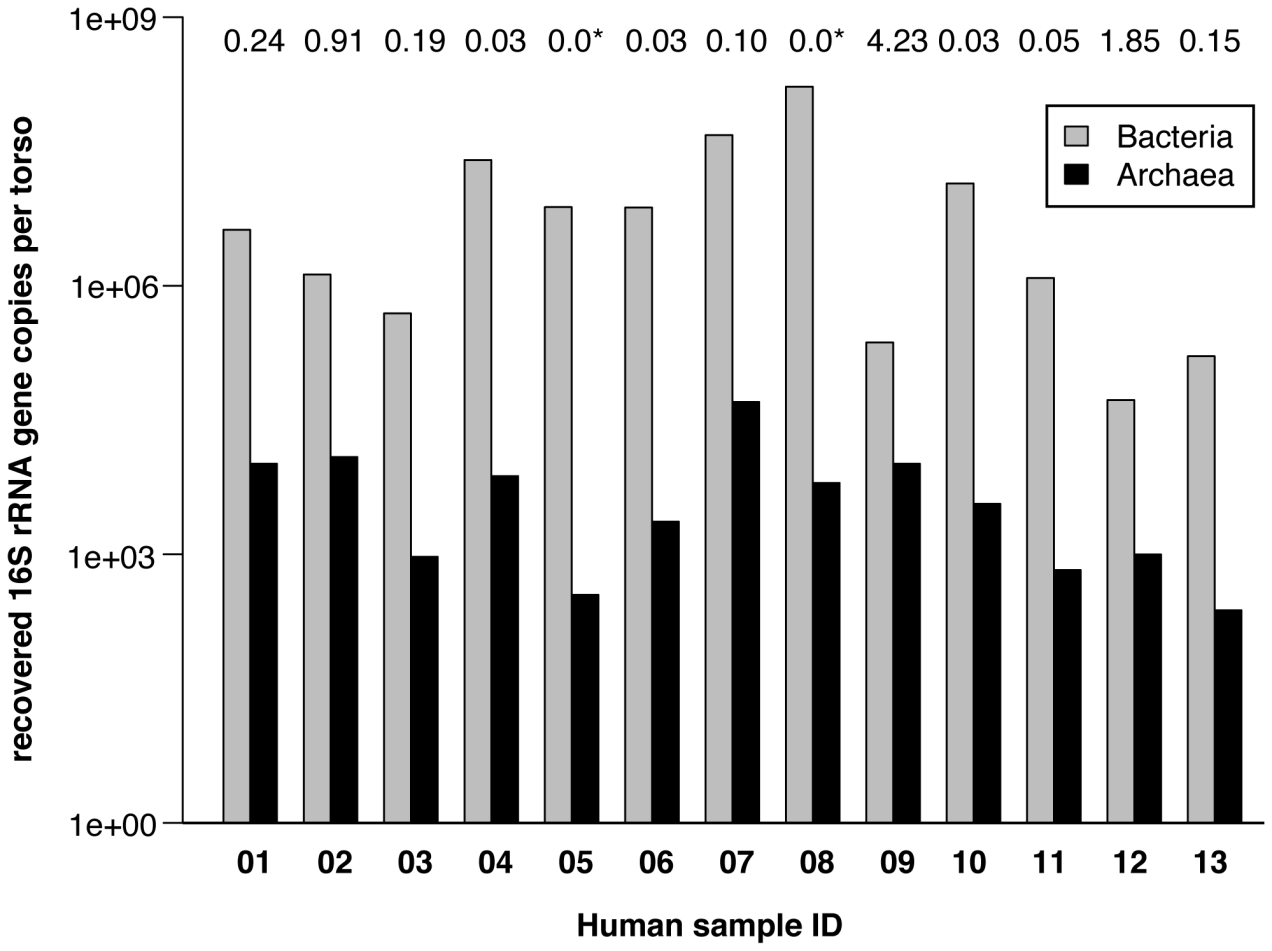
Abundance of bacterial and archaeal 16 S rRNA gene copies retrieved from front torsos of 13 people. Values above bar graphs give percent of archaeal gene copies in the entire prokaryotic microbiome detected. Asterisks indicate an archaeal percentage lower than 0.01. X-axis gives human sample number (Table 1), Y-axis shows log-transformed abundances of 16 S rRNA genes.

In order to investigate the abundance and diversity of Archaea on human skin, wipe-samples from 13 human individuals (7 female and 6 male, age range 20–40, entire front torsos, Table S1) were taken and analyzed with sensitive molecular techniques. All human subjects revealed the presence of archaeal 16 S rRNA genes on their skin, accounting for up to 4.23% of the entire recovered prokaryotic microbiome (0.60% on average, Fig. 1). As Bacteria are likely to possess more ribosomal genes per genome (4.17 on average) compared to Archaea (1.69) [42], the average proportion of archaeal cells could be even greater (1.40% on average, max. 9.86%).

**Figure 2:**
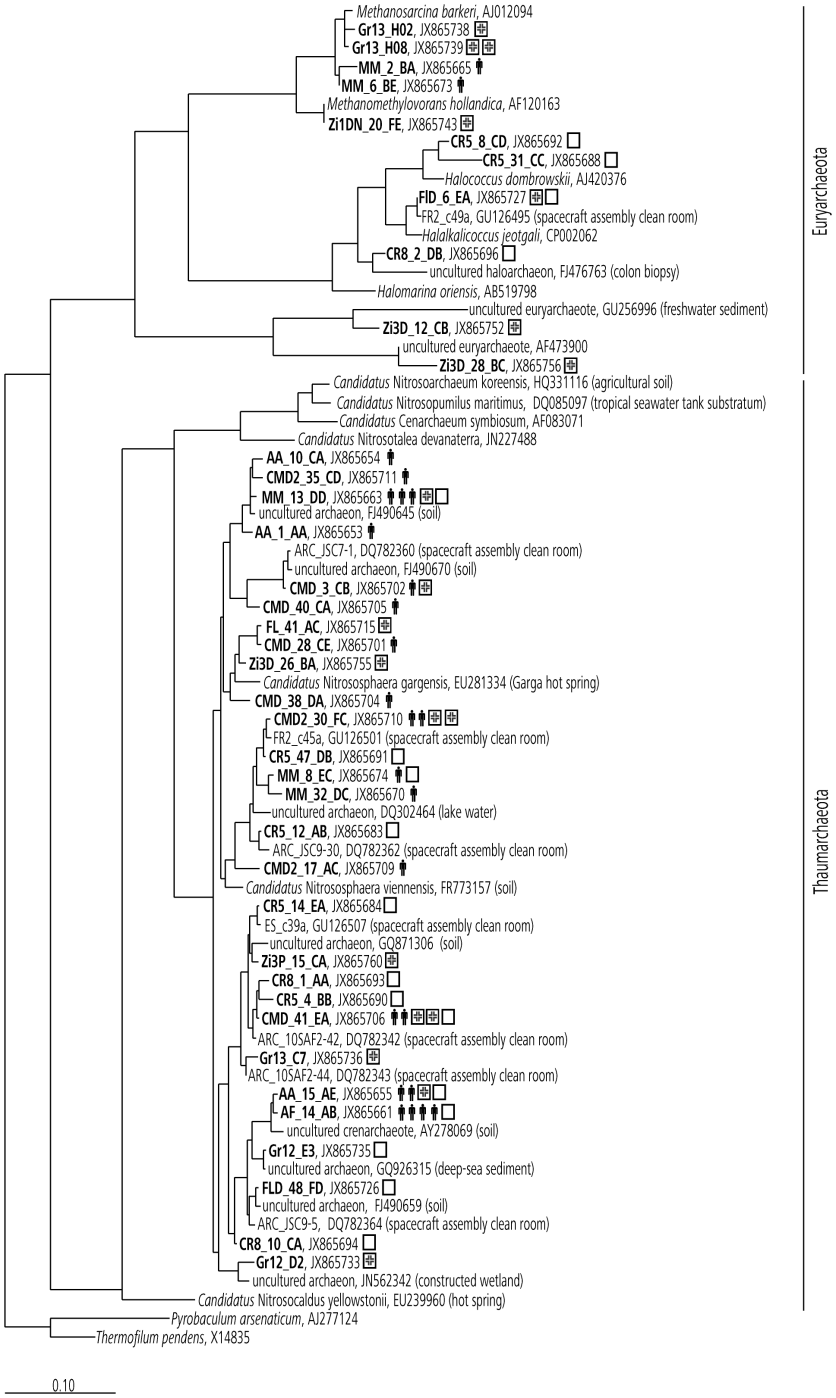
Maximum likelihood tree displaying all detected OTUs from human skin, intensive care unit, and clean room environments. Symbol “*man*”: phylotype retrieved from human skin (the number of symbols gives the number of individuals carrying this phylotype; 5 subjects were screened with respect to the archaeal 16 S rRNA gene pool). Symbol “*hospital*” (square with cross): phylotype detected in intensive care unit (two intensive care units were screened). Symbol “*square*”: detected in a spacecraft assembly clean room (one facility was analyzed). Symbol “*star*” highlights phylotypes that were also found in the propidium monoazide (PMA)-treated sample, i.e. from cells with intact membranes. Scale bar refers to 10% nucleotide substitutions. *Pyrobaculum arsenaticum* and *Thermofilum pendens* (Crenarchaeota) were used as an outgroup.

Five samples were selected for a deeper analysis of the archaeal diversity present on human skin. The archaeal community structure comprised OTUs of the phyla Thaumarchaeota (88% of all OTUs) and Euryarchaeota (12%) with 17 different taxonomic OTUs in total. All human subjects exhibited sequences of Thaumarchaeota. Phylogenetic analysis of these archaeal skin sequences placed them close to ammonia-oxidizing archaea from soil (Thaumarchaota group I.1b, Fig. 2), but interestingly also close to sequences from built environments (clean rooms, intensive care units) found in this and earlier studies [11] (Fig. 2). Besides thaumarchaeal signatures, one human subject revealed two euryarchaeal sequences, which belonged to the taxon *Methanosarcina*, a putative methanogen reported previously for agricultural environments, but also the human intestine [2]. Sequence classifications are summarized in Table 1.

**Table 1:** Analysis of clone libraries and classification of taxonomic OTUs.

	Sample	Hum_01	Hum_04	Hum_07	Hum_08	Hum_10	Graz_12	Graz_13	Rgbg_1	Rgbg_3	Rgbg_F	Rgbg_F	Rgbg_3_ PMA	CR5	CR8	Classification	
	Sample type	Human skin, wipe	Human skin, wipe	Human skin, wipe	Human skin, wipe	Human skin, wipe	ICU, BiSKit	ICU, BiSKit	ICU, wipe	ICU, wipe	ICU, wipe	ICU, wipe	ICU, wipe, PMA	CR ISO5, BiSKit	CR ISO8, BiSKit		
	Detection method	Nested PCR	Nested PCR	Direct PCR	Nested PCR	Nested PCR	Nested PCR	Nested PCR	Direct PCR	Direct PCR	Nested PCR	Direct PCR	Direct PCR	Nested PCR	Nested PCR		
	Coverage	90.5%	100.0%	98.1%	93.8%	97.7%	97.6%	97.9%	100.0%	97.4%	100.0%	97.2%	95.1%	86.5%	94.9%		
OTU ID, Acc. No	# of clones screened	42	46	53	32	44	41	48	45	39	45	36	41	37	39	Phylum	Genus
CR5_31_CC, JX865688		–	–	–	–	–	–	97.92%	–	–	–	2.78%	–	–	–	Euryarchaeota	*Halococcus*
CR8_2_DB, JX865696		–	–	–	12.50%	–	–	–	–	–	–	–	–	–	–	Euryarchaeota	unclassified Halobacteriaceae
CR5_8_CD, JX865692		–	–	–	3.13%	–	–	–	–	–	–	–	–	–	–	Euryarchaeota	unclassified Halobacteriaceae
FlD_6_EA, JX865727		–	–	–	–	–	–	–	–	–	–	8.33%	–	–	–	Euryarchaeota	unclassified Halobacteriaceae
Zi1DN_20_FE, JX865743		–	–	11.32%	–	–	–	–	–	–	–	–	–	–	–	Euryarchaeota	*Methanomethylovorans*
Gr13_H02, JX865738		–	–	-	–	–	–	–	–	7.69%	–	–	–	–	–	Euryarchaeota	*Methanosarcina*
Gr13_H08, JX865739		–	–	3.77%	–	–	–	–	–	–	–	–	–	–	–	Euryarchaeota	*Methanosarcina*
MM_2_BA, JX865665		–	–	–	–	–	–	–	–	15.38%	–	–	–	–	–	Euryarchaeota	*Methanosarcina*
MM_6_BE, JX865673		–	–	–	–	–	–	–	8.89%	–	–	–	–	–	–	Euryarchaeota	*Methanosarcina*
Zi3D_28_BC, JX865756		–	–	–	–	–	–	–	–	–	–	–	–	–	51.28%	Euryarchaeota	unclassified Euryarchaeota
CMD_28_CE, JX865701		–	–	1.89%	–	–	–	–	–	–	–	–	–	–	–	Thaumarchaeota	*Candidatus* Nitrososphaera
FL_41_AC, JX865715		–	–	–	–	–	–	–	–	–	–	–	2.44%	–	–	Thaumarchaeota	*Candidatus* Nitrososphaera
Zi3D_26_BA, JX865755		–	–	–	–	–	–	–	–	–	–	–	–	–	30.77%	Thaumarchaeota	*Candidatus* Nitrososphaera
AA_15_AE, JX865655		2.38%	–	–	–	2.27%	–	–	42.22%	–	–	22.22%	51.22%	–	2.56%	Thaumarchaeota	*Candidatus* Nitrososphaera
AF_14_AB, JX865661		2.38%	–	9.43%	34.38%	–	–	–	44.44%	5.13%	–	–	–	2.70%	–	Thaumarchaeota	*Candidatus* Nitrososphaera
CMD_41_EA, JX865706		–	26.09%	28.30%	–	–	2.44%	–	–	64.10%	-	33.33%	19.51%	10.81%	2.56%	Thaumarchaeota	*Candidatus* Nitrososphaera
CR5_14_EA, JX865684		–	–	–	–	–	–	–	–	–	100.00%	-	19.51%	–	–	Thaumarchaeota	*Candidatus* Nitrososphaera
CR5_4_BB, JX865690		–	–	–	–	–	21.95%	–	–	–	–	–	–	–	–	Thaumarchaeota	*Candidatus* Nitrososphaera
CR8_1_AA, JX865693		–	–	–	3.13%	–	–	–	–	–	–	–	–	–	–	Thaumarchaeota	*Candidatus* Nitrososphaera
CR8_10_CA, JX865694		–	–	–	12.50%	–	–	–	–	–	–	–	–	–	–	Thaumarchaeota	*Candidatus* Nitrososphaera
FLD_48_FD, JX865726		–	–	3.77%	-	–	–	–	–	–	–	–	–	–	–	Thaumarchaeota	*Candidatus* Nitrososphaera
Gr12_E3, JX865735		–	–	–	–	–	–	–	–	–	–	–	–	5.41%	–	Thaumarchaeota	*Candidatus* Nitrososphaera
Gr13_C7, JX865736		–	–	5.66%	–	–	–	–	–	–	–	–	–	–	–	Thaumarchaeota	*Candidatus* Nitrososphaera
Zi3P_15_CA, JX865760		–	–	–	–	–	68.29%	–	–	–	–	–	–	–	–	Thaumarchaeota	*Candidatus* Nitrososphaera
CMD_3_CB, JX865702		–	–	22.64%	–	–	–	–	–	–	–	19.44%	4.88%	–	–	Thaumarchaeota	*Candidatus* Nitrososphaera
CMD_40_CA, JX865705		–	–	–	–	–	–	–	–	–	–	–	–	2.70%	–	Thaumarchaeota	*Candidatus* Nitrososphaera
AA_1_AA, JX865653		2.38%	–	–	–	–	–	–	–	–	–	–	–	–	–	Thaumarchaeota	*Candidatus* Nitrososphaera
AA_10_CA, JX865654		2.38%	–	–	–	–	–	–	–	–	–	–	–	–	–	Thaumarchaeota	*Candidatus* Nitrososphaera
CMD2_17_AC, JX865709		–	–	–	–	–	–	–	–	–	–	–	–	8.11%	–	Thaumarchaeota	*Candidatus* Nitrososphaera
CMD2_30_FC, JX865710		–	–	13.21%	28.13%	–	7.32%	–	4.44%	2.56%	–	–	2.44%	–	–	Thaumarchaeota	*Candidatus* Nitrososphaera
CMD2_35_CD, JX865711		–	–	–	–	–	–	–	–	–	–	–	–	2.70%	–	Thaumarchaeota	*Candidatus* Nitrososphaera
CR5_12_AB, JX865683		–	–	–	–	–	–	–	–	–	–	–	–	2.70%	–	Thaumarchaeota	*Candidatus* Nitrososphaera
CR5_47_DB, JX865691		–	–	–	–	–	–	2.08%	–	–	–	–	–	–	–	Thaumarchaeota	*Candidatus* Nitrososphaera
MM_13_DD, JX865663		90.48%	73.91%	-	6.25%	97.73%	–	–	–	–	–	–	–	37.84%	–	Thaumarchaeota	*Candidatus* Nitrososphaera
MM_8_EC, JX865674		–	–	–	–	–	–	–	–	–	–	13.89%	–	8.11%	–	Thaumarchaeota	*Candidatus* Nitrososphaera
CMD_38_DA, JX865704		–	–	–	–	–	–	–	–	–	–	–	–	2.70%	–	Thaumarchaeota	*Candidatus* Nitrososphaera
Gr12_D2, JX865733		–	–	–	–	–	–	–	–	5.13%	–	–	–	–	–	Thaumarchaeota	*Candidatus* Nitrososphaera
MM_32_DC, JX865670		–	–	–	–	–	–	–	–	–	–	–	–	–	12.82%	Thaumarchaeota	*Candidatus* Nitrososphaera
Zi3D_12_CB, JX865752		–	–	–	–	–	–	–	–	–	–	–	–	16.22%	-	unclassified Archaea	unclassified Archaea

Abbreviations: ICU (intensive care units); CR (clean room).doi:10.1371/journal.pone.0065388.t001

**Figure 3:**
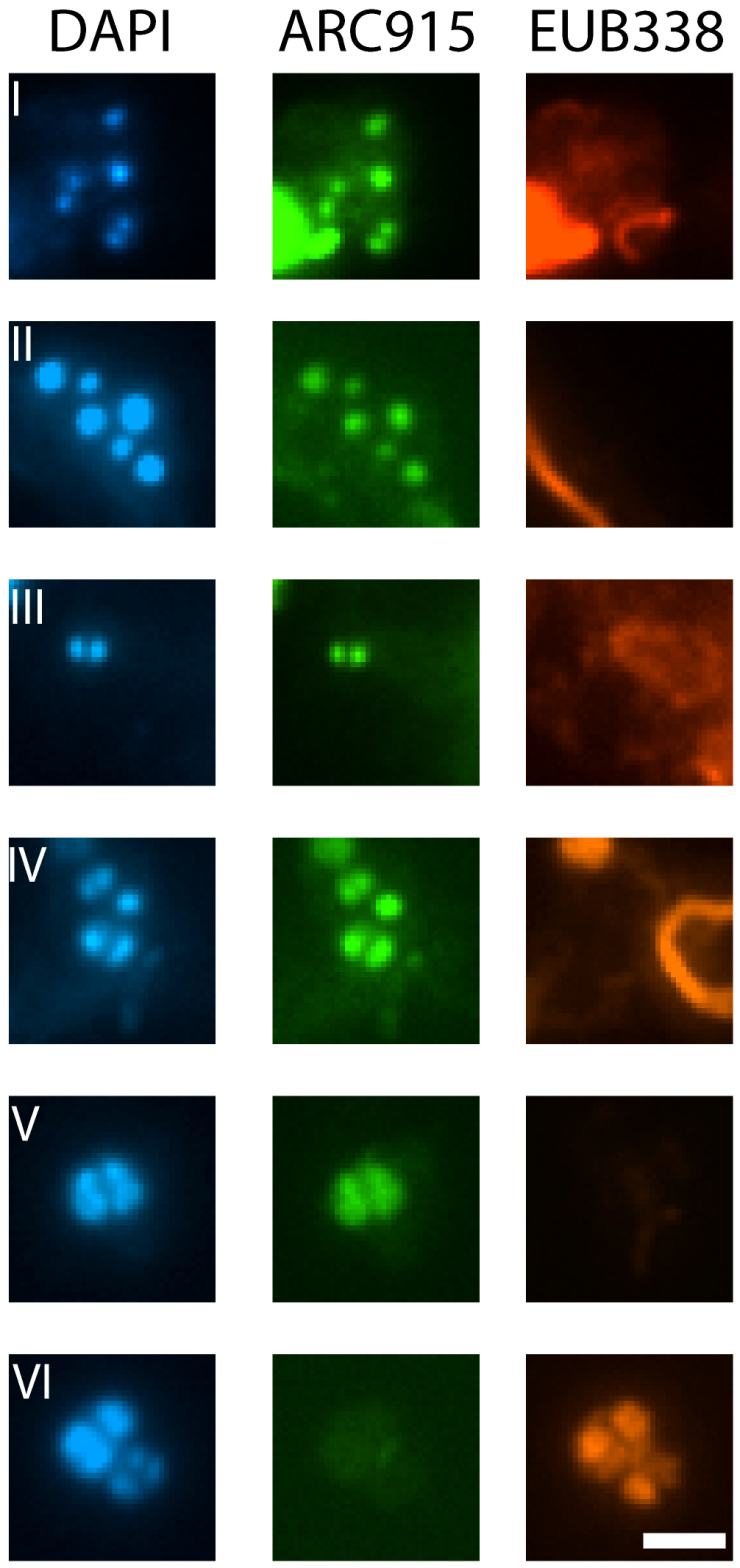
Fluorescence *in situ* hybridization, performed on a human skin wipe-sample for visualization of Archaea. DNA-containing cell (DAPI stain): blue, Archaea: green, Bacteria: red. I-V: Examples of positive archaeal signals (small cocci, probe ARC915 labeled with rhodamine green) are shown, which give a positive signal with DAPI and no signal with the Bacteria-directed probe (EUB338/I labeled with CY3). VI: Example of a positive bacterial signal. Bar: 2 µm.

The presence of Archaea on human skin was further confirmed by fluorescence *in situ* hybridization experiments (Fig. 3). Unfortunately, the relative abundance of archaeal compared to bacterial cells could not be calculated due to the high amount of fluorescence-active particulates and fibers in the specimens (see Material and Methods for details), but archaeal signals were obvious and easily detectable. Archaeal cells were visualized as small cocci (approx. 0.5 µm in diameter) in the skin microbiome. Their shape and size was similar to thaumarchaeal cells previously detected in sludge samples [43].

Because human-dominated environments reflect the microbial diversity associated with the human skin and body [44], we can now propose a logical reason for the earlier discovery of archaeal signatures in controlled clean room facilities around the world [10], [11], [13]. The presence of (thaum-) archaeal signatures in clean rooms was confirmed in this study (EADS clean room facility) and further expanded to hospitals. Two intensive care units were sampled and the presence of Thaumarchaeota in these artificial environments was affirmed (Fig. 2). So far, all man-made environments studied by the authors have revealed the presence of archaeal 16 S rRNA genes, belonging to two different phyla, Thaumarchaeota and Euryarchaeota, which can be attributed to the sensitive and appropriate assays employed. The integrity and therefore probable viability of archaeal cells in floor wipe-samples were also proven by FISH [11] and with a molecular-based viability assay (Table S1, Fig. 2). The clean room euryarchaeota included methanogens and different halophiles; both groups have been associated with human mouth and intestinal flora [2], [8], and were recently also found in navels [5]. However, the aforementioned study on navels did not reveal any thaumarchaeal signatures and only three euryarchaeal phylotypes in a very small subset of the samples [6]. Euryarchaeota (again methanogens and halophiles), Crenarchaeota and Thaumarchaeota were detected in a study by Caporaso *et al.*, 2011, who analyzed the microbial community of human gut, tongue and palms of only two individuals but at 396 timepoints. In particular, the palm microbiome revealed the (fluctuating) presence of *Nitrososphaera* related sequences. Based on their statistical analysis, the authors considered those organisms insignificant and transient members of the human microbiome. Certainly, the palm skin represents one of the major contact surfaces of humans to their biotic and abiotic environment, and transient microorganisms can be found there more than elsewhere on human skin. Consequently, we excluded human palms and focused on human torso skin, which might harbor a more typical, less influenced microbial diversity. We argue based on our results of 13 human skin samples, that Caporaso *et al.* may have underestimated the importance of Archaea and particularly Thaumarchaeota on human skin. To emphasize the finding of a general presence of Archaea on human skin (all samples revealed archaeal signatures), we were even able to visualize archaeal cells, indicating their active physiological status and their obvious presence in samples from human skin. We can conclude that previous studies have either (methodically) overlooked the archaeal diversity associated with human skin or underestimated their abundance.

Our study was the first to systematically show that Archaea, in particular Thaumarchaeota, are consistently present on human skin. This finding leads to a number of questions that cannot be answered at the current status of knowledge. For instance, the role, metabolism, infestation rate or also the origin of archaea associated with skin are unclear to date and need to be tackled in subsequent studies.

**Figure 4:**
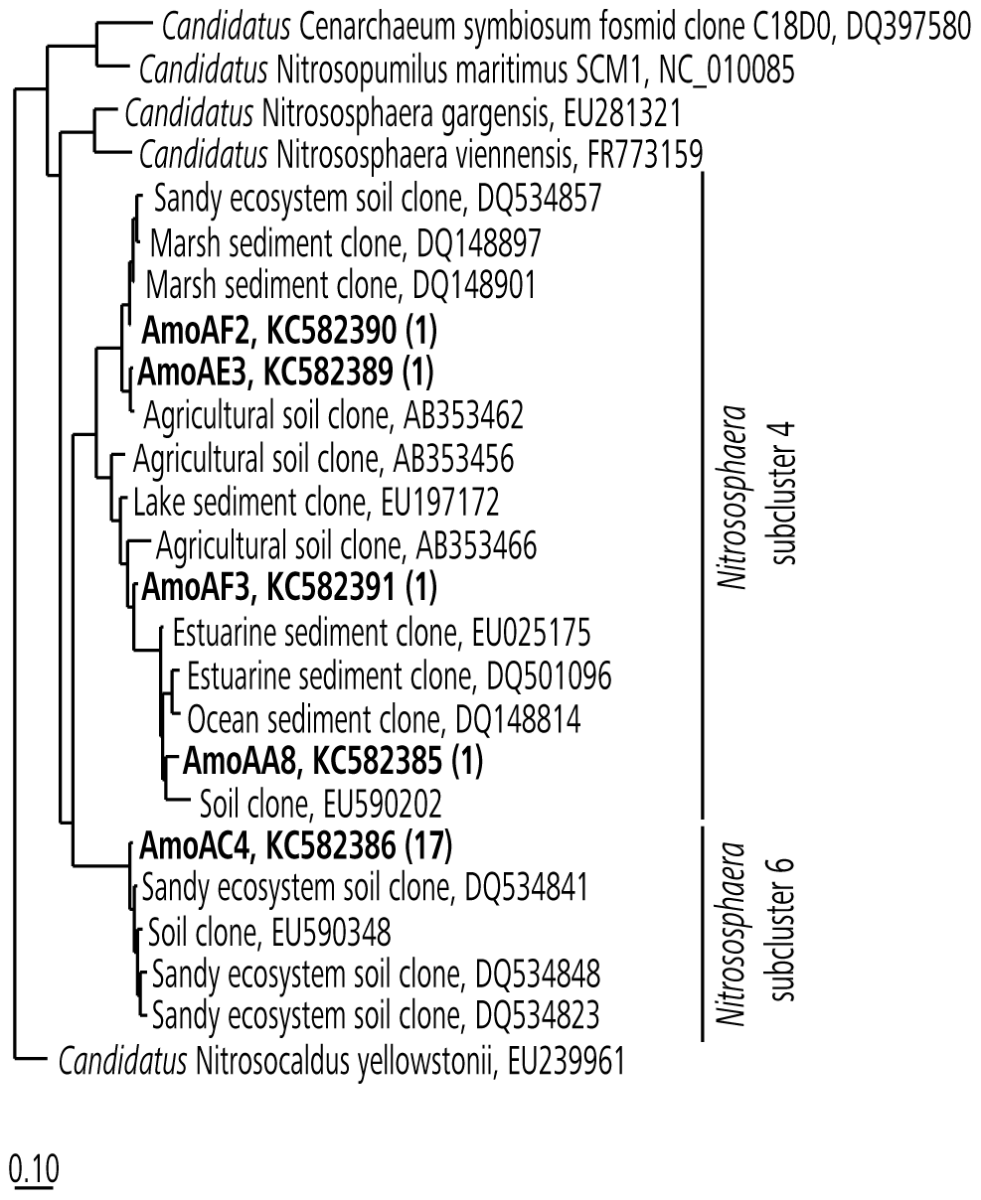
Maximum likelihood tree based on archaeal *amoA* gene sequences. Sequences recovered in this study are shown in bold. Information in parenthesis gives the number of retrieved sequences. Bar refers to 10% nucleotide substitutions per site.

Interestingly, representatives of Thaumarchaeota cluster I.1b, were found in soil and aquatic environments, but also in wastewater treatment plants, as reported recently [43], [45]. The detection of such thaumarchaeal sequences and cells on human skin and engineered environments could point to novel, currently unknown roles and metabolic capabilities besides chemolithoautotrophy [43]. However, we note that all so far cultivated thaumarchaeal species are ammonia oxidizers [12], [39], [46], [47] and the human skin is constantly emanating low amounts of ammonia [48]. We were able to amplify and sequence *amoA* genes from a pooled sample of all 13 human subjects. Megablast analyses against the NCBI nucleotide collection showed clearly that these sequences belong to *amoA* genes (E-value = 0). A phylogenetic tree of the retrieved *amoA* sequences is depicted in Figure 4. The sequences obtained were not closely related to *Nitrososphaera viennensis* or *N. gargensis amoA* genes, but were affiliated to two *Nitrososphaera* subcluster (4 and 6, Fig. 4), similar to the majority of the 16 S rRNA genes (Fig. 2).

This finding suggests at least one possible explanation for the presence of these microorganisms. It can be hypothesized, that a chemolithotrophic ammonia turnover by Thaumarchaeota could influence the pH regulation of the human skin and therefore the natural protective layer, but this remains to be proven. Additionally, the interaction of humans with Archaea seems not to be restricted to a passive and/or indirect methanogenic activity in colon and mouth cavity, but could be an active and direct relationship. Because protocols as used for the Human Microbiome Project apparently underestimated the presence of Archaea in general, screening methods in science and medicine should be better geared towards improved detection of Archaea, which will then lead to a comprehensive understanding of their beneficial or potentially pathogenic role in the human (skin) microbiome.

## Supporting Information

Table S1 Overview of samples, characteristics and detection of Archaea.
(DOCX)
