# Supplementary material for: Archaea on Human Skin
Source: PLoS One. 2013 Jun 12;8(6):e65388. doi: 10.1371/journal.pone.0065388 (PMC3680501; doi:10.1371/journal.pone.0065388)
Supplement: Table S1 — Overview of samples, characteristics and detection of Archaea. (DOCX) [file pone.0065388.s001.docx]

Supporting Information: Table S1: Overview of samples, characteristics and detection of Archaea.

| **Sample** | **Sample type** | | **QPCR** | | | | | | | **Direct PCR** | | **Nested PCR** | | **FISH** |
| --- | --- | --- | --- | --- | --- | --- | --- | --- | --- | --- | --- | --- | --- | --- |
|  |  |  | **Bacterial 16S rRNA gene copies per torso or m² floor** | **Bacterial 16S rRNA gene copies: standard deviation** | **Archaeal 16S rRNA gene copies per torso or m² floor** | | **Archaea 16S rRNA gene copies: standard deviation** | | **Percent archaeal 16S rRNA gene copies** | **Archaeal 16S rRNA gene PCR** | **Archaeal 16S rRNA gene library: clones/coverage^1^** | **Archaeal 16S rRNA gene nested-PCR** | **Archaeal 16S rRNA gene library (nested): clones/coverage^1^** |  |
| Hum_01 | Human skin, wipe | | 4,214,834 | 10% | 10,268 | | 12% | | 0.24% | negative | nd | **positive** | 42 / 90.5 | nd |
| Hum_02 | Human skin, wipe | | 1,334,029 | 4% | 12,223 | | 21% | | 0.91% | **positive** | nd | negative | nd | nd |
| Hum_03 | Human skin, wipe | | 491,439 | 11% | 938 | | 31% | | 0.19% | nd | nd | nd | nd | nd |
| Hum_04 | Human skin, wipe | | 25,363,739 | 48% | 7,488 | | 8% | | 0.03% | negative | nd | **positive** | 46 / 100 | nd |
| Hum_05 | Human skin, wipe | | 7,557,334 | 10% | 353 | | 64% | | 0.00% | nd | nd | nd | nd | nd |
| Hum_06 | Human skin, wipe | | 7,474,329 | 18% | 2,318 | | 21% | | 0.03% | negative | nd | negative | nd | nd |
| Hum_07 | Human skin, wipe | | 48,120,134 | 3% | 50,248 | | 7% | | 0.10% | **positive** | 53 / 98.1 | **positive** | nd | **positive** |
| Hum_08 | Human skin, wipe | | 167,256,499 | 6% | 6,278 | | 21% | | 0.00% | negative | nd | **positive** | 32 / 93.8 | nd |
| Hum_09 | Human skin, wipe | | 232,734 | 8% | 10,288 | | 1% | | 4.23% | nd | nd | nd | nd | nd |
| Hum_10 | Human skin, wipe | | 13,886,809 | 17% | 3,663 | | 13% | | 0.03% | negative | nd | **positive** | 44 / 97.7 | nd |
| Hum_11 | Human skin, wipe | | 1,218,714 | 16% | 668 | | 79% | | 0.05% | nd | nd | nd | nd | nd |
| Hum_12 | Human skin, wipe | | 52,859 | 21% | 998 | | 86% | | 1.85% | nd | nd | nd | nd | nd |
| Hum_13 | Human skin, wipe | | 163,199 | 11% | 238 | | 173% | | 0.15% | nd | nd | nd | nd | nd |
| Graz_12 | Intensive care unit, BiSKit | | nd | nd | 14,228 | | 28% | | nd | nd | nd | **positive** | 41 / 97.6 | nd |
| Graz_13 | Intensive care unit, BiSKit | | nd | nd | 1,112 | | 63% | | nd | nd | nd | **positive** | 48 / 97.9 | nd |
| Rgbg_1 | Intensive care unit, wipe | | 69,296 | 10% | 2,043 | | 16% | | 2.86% | **positive** | 45 / 100 | **positive** | nd | nd |
| Rgbg_3 | Intensive care unit, wipe | | 153,381 | 12% | 6,581 | | 8% | | 4.11% | **positive** | 39 / 97.4 | **positive** | nd | nd |
| Rgbg_F | Intensive care unit, wipe | | 62,180 | 12% | 2,498 | | 17% | | 3.86% | **positive** | 36 / 97.2 | **positive** | 45 /100 | nd |
| Rgbg_1_PMA | Intensive care unit, wipe, PMA^2^ | | 65 | 19% | BDL | | BDL | | BDL | negative | nd | negative | nd | nd |
| Rgbg_3_PMA | Intensive care unit, wipe, PMA^2^ | | 24,730 | 24% | 2,067 | | 6% | | 7.71% | **positive** | 41 / 95.1 | **positive** | nd | nd |
| Rgbg_F_PMA | Intensive care unit, wipe, PMA^2^ | | 515 | 31% | BDL | | BDL | | BDL | negative | nd | negative | nd | nd |
| CR5 | Clean room ISO 5, BiSKit | | 6,323,082 | 5% | 167,178 | | 12% | | 2.58% | negative | nd | **positive** | 37 / 86.5 | nd |
| CR8 | Clean room ISO 8, BiSKit | | 2,707,996 | 2% | 92,074 | | 16% | | 3.29% | negative | nd | **positive** | 39 / 94.9 | nd |
| ^1^clones / coverage give the number of clones analyzed and the percent coverage after OTU grouping at 1% level | | | | | | | | | | |  |  |  |  |
| ^2^PMA = sample treated with propidium monoazide | | | |  |  |  | |  | |  |  |  |  |  |
| nd = not determined | |  | |  |  |  | |  | |  |  |  |  |  |
| BDL = Below Detection Limit of qPCR, see Methods for details | | | |  |  |  | |  | |  |  |  |  |  |
